# Photoluminescent behavior and structural analysis of SnO layers formed by laser-induced oxidation

**DOI:** 10.1080/14686996.2025.2450213

**Published:** 2025-01-08

**Authors:** Leonid Fedorenko, Patrik Ščajev, Saulius Miasojedovas, Vidas Pakštas, Vitalija Jasulaitienė, Gediminas Kreiza, Pavels Onufrijevs, Volodymyr Yukhymchuk, Evgen Soloviev, Hidenori Mimura

**Affiliations:** a V. Lashkaryov Institute of Semiconductor Physics, National Academy of Sciences of Ukraine, Kyiv, Ukraine; b Institute of Photonics and Nanotechnology, Vilnius University, Vilnius, Lithuania; c Department of Characterization of Materials Structure, Center for Physical Sciences and Technology, Vilnius, Lithuania; d Institute of Technical Physics, Faculty of Natural Sciences and Technology, Riga Technical University, Riga, Latvia; e Research Institute of Electronics, Shizuoka University, Hamamatsu, Japan

**Keywords:** SnO monoxide layer, laser nanotechnology, photoluminescence femtosecond diagnostics, excitons, nanophotonics, optoelectronics

## Abstract

We develop a rapid and spatially controlled formation method of a smooth polycrystalline SnO film preventing the transition to a more stable SnO_2_ phase. The phase and structural state of a SnO oxide film, which was formed by pulsed irradiation of a Nd:YAG laser on a tin plate in contact with air and distilled water, were studied. XRD, Raman spectra, and kinetics of the exciton PL under femtosecond excitation showed a more perfect textured structure and strong exciton emission of the SnO film obtained by the laser under the conditions of Sn contact with air. The obtained results indicate the applicability of the laser method for the formation of SnO layers, according to the given topology, which can be used for UV-emitting devices and photocatalysts.

## IMPACT STATEMENT

Polycrystalline smooth SnO layers, obtained by laser irradiation, consist of well-fused nanocrystals with the possibility of forming microtopography. Laser formation of the SnO film prevents the transition to the SnO_2_ phase.

## GRAPHICAL ABSTRACT

**Figure:**
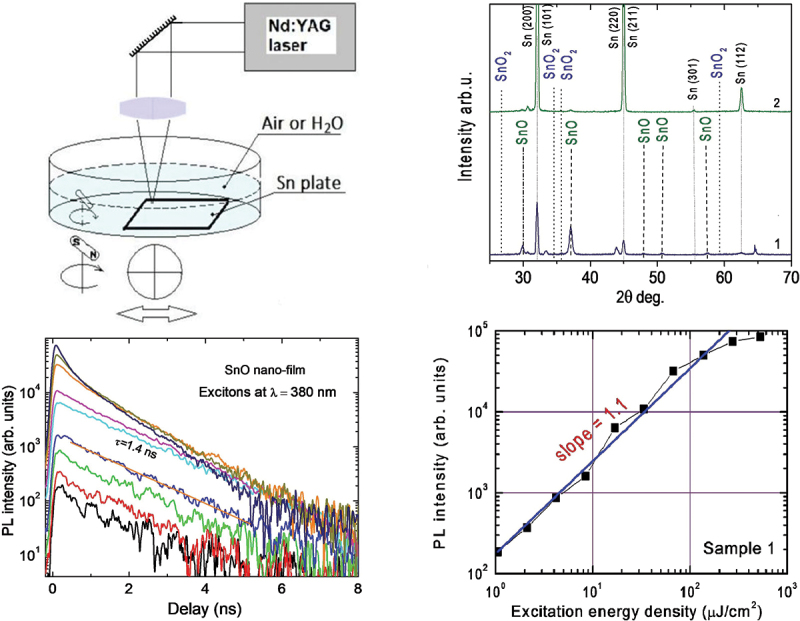

## 1. Introduction

The study of the emission properties of metal oxide layers of submicron and nanoscale sizes, as well as methods of their formation, is motivated by the progress of nanophononics in terms of the development of new photonic media for the needs of modern optoelectronics. A promising class of materials are tin oxides, in particular, SnO and SnO_2_, with a wide variation in band gap Е_g_: SnO is a semiconductor with an indirect band gap of E_g_ = 0.7 eV and an optical gap E_g_ = 2.7–3.4 eV [1,2] and direct band gap (E_g_ = 3.6 eV) for SnO_2_ [3]. Tin monoxide (SnO – Sn^+2^) and tin dioxide (SnO_2_ - Sn^+4^) are p-type and n-type semiconductors, respectively. They have been successfully used in optoelectronics, energy storage devices, and sensors. SnO, as a semiconductor, has a separate request for application: supercapacitors [4,5], electrodes for lithium-ion batteries [6], gas sensors [7], and catalysts and photocatalysts [8,9]. However, during the formation of SnO nanolayers, a problem arises due to the less stable phase of SnO (Sn^+2^) compared to SnO_2_ (Sn^+4^) due to the favourable oxidation of Sn^+2^. To avoid the latter, micro- and nanocrystalline SnO particles are produced using chemical-physical processes, such as electrochemical, ultrasonic, hydrothermal, and microwave methods, using ionic liquids, and physical vacuum deposition (PVD) [10]. In addition, organic additives, surfactants, an inert atmosphere (Ar or N_2_) and reducing (H_2_) gases are used to increase the yield of the lower valence phase [10]. It should be noted that the use of PVD requires the creation of a SnO ceramic target, which is a very difficult task, since it always leads to impurity phases of SnO_2_ either metallic Sn [11] as target material for depositing SnO thin films. Moreover, existing methods of forming SnO nanolayers and nanoparticles often do not satisfy the necessary variations in the flexible express nanotechnologies. The contact medium in the zone of laser action (air, distilled water) was varied for reasons of stimulation of oxidation. It is well known that the concentration of oxygen atoms in air under optimal conditions is ~0.4 g/m^3^, and in water ~5 g/m^3^. An additional factor that could also increase the oxidation of tin in contact with water could be large temperature and pressure gradients due to the cumulative evaporation of water in the laser pulse action zone. The above-mentioned stimulated our intention to form SnO nanofilms by direct laser-stimulated oxidation of a Sn metal plate by a pulsed Nd:YAG laser according to the given topology in contact with air and distilled water.

## 2. Experimental

The formation of tin oxide nanolayers was performed on the surface of the β-Sn plates manufactured from Sn with 99.5% purity (American Elements, SN-M-025-PL). The plates were irradiated with pulses (τ_p_ = 0.150 ms) of the Nd:YAG laser model NL301G manufactured by EKSPLA (Lithuania) at a wavelength of 1064 nm in software scanning mode with the ability to focus the beam, adjust the illumination, the frequency of repetition of laser pulses and the degree of overlap of laser spots to achieve the greatest homogeneity of the light flux. Deionized water and air at normal conditions were selected as the environment in the active technological zone based on the comparison of the estimated concentrations of the free oxygen atoms (N_O_) in the deionized agitated water and in the air. It is known that oxygen atoms consist of N_O_ ~ 0.9% of the concentration of all molecules in water N_H2O_ = 3.35 × 10^22^ cm^−3^, i.e. N_O_ ≈ 3.0 × 10^20^cm^−3^. The part of atomic oxygen in normal conditions in the air is 23% of the concentration of air molecules N_air_ ≈ 2.7 × 10^19^cm^−3^, i.e. N_O_ = 0.23 × 2.7 × 10^19^cm^−3^ = 6.21 × 10^18^cm^−3^. That is, in the case of water, as in the environment, a significant effect could be expected.

Photoluminescence (PL) spectra and PL relaxation spectra were measured by a femtosecond laser PHAROS (Light Conversion, Lithuania) excitation (τ_p_ = 180 fs) at a wavelength of λ = 266 nm with a regulated excitation pulse fluence in the 1–274 µJ/cm^2^ range. The PL spectra were measured with a streak camera (Hamamatsu C10627, Japan) achieving 20 ps temporal resolution.

The structural properties and phase composition of the films were studied by Raman spectroscopy with a Renishaw In-ViaV727 (UK) spectrometer with a backscattering geometry at room temperature. The phonon excitation was induced with an Ar^+^- laser (λ_ex_ = 514.5 nm).

The XRD structural characterization was performed by the Rigaku, SmartLab diffractometer (Japan) with a 9 kW Cu rotating anode X-ray tube (Cu Kα radiation) and scintillation detector SC-70.

The high-resolution XPS valence band (VB) spectra of the SnO sample were analysed by using Kratos AXIS Supra+ spectrometer (UK) with monochromatic Al Kα (1486.6 eV) X-ray radiation powered at 225 W. The base pressure in the analysis chamber was less than 1 × 10^−9^ mbar and a low electron flood gun was used as a charge neutralizer. The depth profiles were obtained by ion etching of the films with 500 eV Ar^+^ ions and a current density of 3.7 μA/cm^2^. The binding energy scale was calibrated by setting the adventitious carbon peak at 284.8 eV. XPS data was converted to VAMAS format and processed using the Avantage v5.9922 software (Thermo Scientific, East Grinstead, UK).

Surface quality and cross section were verified by SEM measurements (Helios NanoLab 650, FEI, USA). AFM scans were performed using the Bruker Dimension Icon (USA) microscope.

The samples’ absorbance was determined by measuring the total reflection in an integrating sphere spectrometer (Perkin Elmer Lambda 950, USA).

## 3. Results and discussion

The details of the sample production are described in Table 1. Air and water were used as mediums for the laser irradiation. Irradiation levels (laser pulse fluence – F) were selected close to but not exceeding the microablation threshold. The microablation threshold of the tin plate when exposed to laser action, in particular, in conditions of contact with air, showed a value of approximately 1.3 J/cm^2^, which, when exposed to pulses of free generation of a Nd^+3^:YAG laser (t_p_ = 150 µs), is equivalent to an intensity of I = 8.5 kW/cm^2^.

**Table 1.** SnO layers produced at different laser processing conditions.

Series of samples No.	Process details		(002) SnO XRD peak intensity (cps)	Exciton peak intensity (arb.un.)	Exciton lifetime (ns)
	F, J/cm^2^	Environment			
(1)	1.27	Air	1580	3700	1.4
(2)	1.27	Water	75	–	–

**Figure 1.**
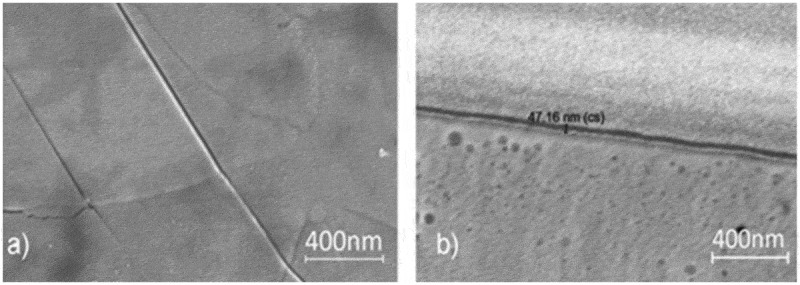
SEM top (a) and side (b) view of the best morphology layer (sample 1).

Figure 1 presents SEM images illustrating: a) the submicron size range of the polycrystalline textured sample and b) the thickness of the SnO film, approximately 50 nm.

**Figure 2.**
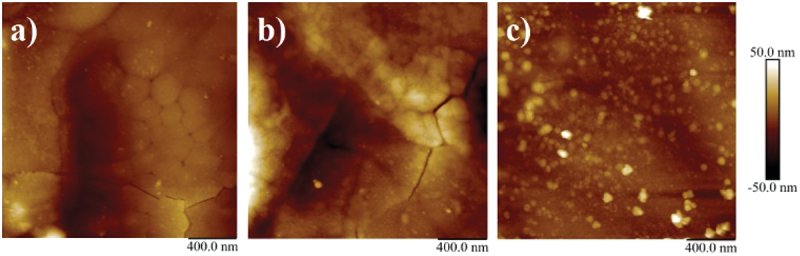
AFM images of different fractures of the SnO films from series (1) with roughness RMS: a) −13.1 nm, b) − 8.82 nm, c) − 7.63 nm.

AFM results for the SnO nanofilm fractures, of series 1, are shown in Figure 2. The roughness is as low as 10 nm.

**Figure 3.**
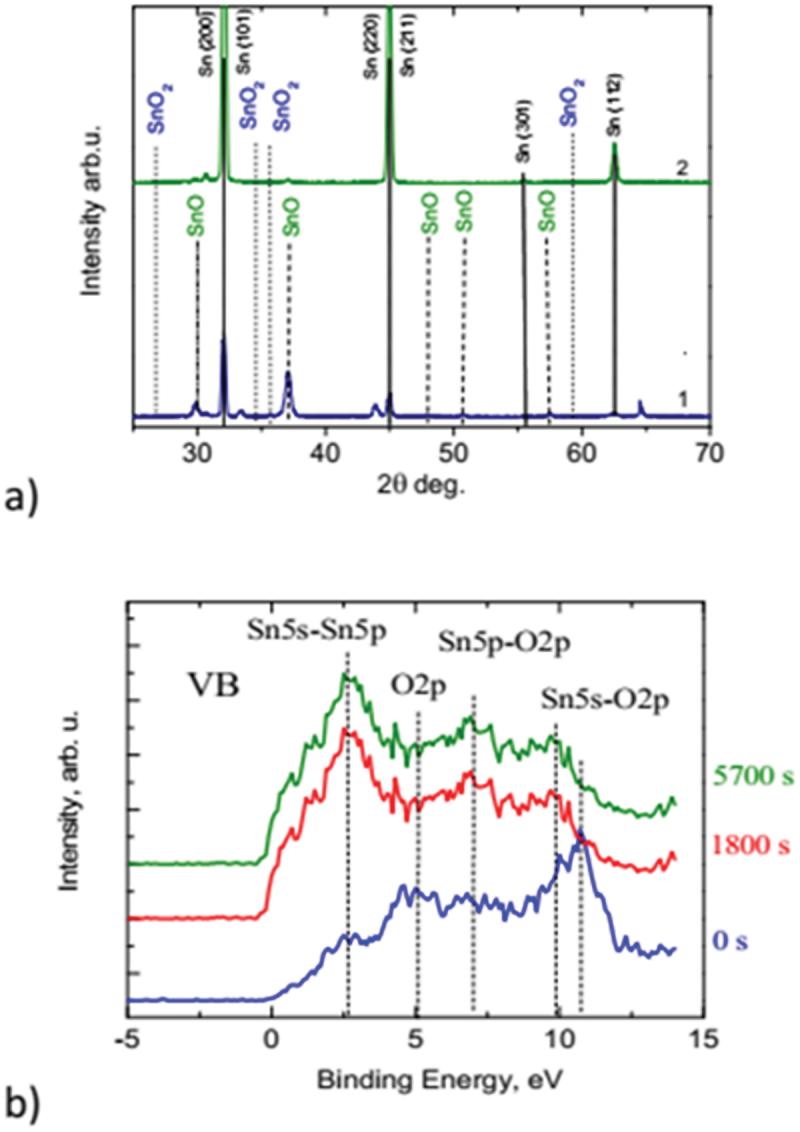
a) XRD. X-ray diffractograms from the SnO layers, formed by pulsed laser processing of the Sn surface at air conditions (1) and in water (2). Sn, SnO, and SnO_2_ peaks are identified. b) the valence band spectrum obtained by XPS before the ion sputtering (0 s) and after 1800 s, 5700 s ion sputtering.

After surface treatment of the samples by laser radiation (in air/water), XRD structural studies were performed. The X-ray diffractograms of the SnO layers formed on the Sn surface (Figure 3) show strong peaks at 2Ɵ = 29.87°, 33.33°, 37.11°, 44.34°, 47.85°, 50.73°, and 57.02° which corresponds to the peak of the (101), (110), (002), (102), (200), (112), and (003) planes, accordingly, crystalline tetragonal structure SnO (ICDD # 04-005-4540), formed on Sn (ICDD # 00-004-0673).

The XRD analysis of the obtained layers in Figure 3 shows that air provides the better way for oxidation, as only samples of the one series in air (1) show considerably large XRD peaks for the SnO phase. In Table 1, the intensities of SnO (101) and SnO (002) diffraction peaks were compared for the series of the samples obtained in air and water environments. SnO diffraction peaks from a series of samples in air were obtained 20 times more intense than of samples in water, series (2), indicating that air provides a better way for oxidation. AFM images provided good smoothness.

By comparing the laser-assisted SnO film formation in our experiment with the evolution stages of the quasi-equilibrium thermal annealing process [12], we can conclude that the finishing phase stage of a laser action in our case corresponds to the thermal annealing process when the temperature does not exceed 550 C^o^. Peak at 33°, possibly can be identified with an intermediate stoichiometry between SnO and SnO_2_, e.g. Sn_2_O_3_. The XRD analysis of the obtained layers in Figure 3. XRD shows that only sample 1 shows considerably large XRD peaks for the SnO phase. The size L of the crystalline component of the laser-formed film can be estimated using the Scherrer equation:

(1) $$B\left(2\theta \right)=\frac{\mathrm{K\lambda}}{L\cdot \mathrm{cos\theta}}$$

where B(2θ) is the peak width, θ is the diffraction angle at a certain value of 2θ, λ is the wavelength of X-ray radiation, and the constant K is a function of crystallite shape, but is usually taken to be approximately 1.0 for spherical particles. Taking 2θ = 37 degrees, cos (θ) = 0.95, λ = 0.154 nm, we get L = (18.6 ± 1.4) nm using the Halder–Wagner Method. Such an unexpected result that air provides a better way of oxidation than water can be explained precisely by the acceleration of oxidation, which at the same fluences in the case of contact with water can accelerate the oxidation transition from SnO to SnO_2_, as shown by XRD, and increase the instability of the process.

XPS surface studies have shown that the Sn sample, treated with various laser excitation energy density, forms SnO and SnO_2_ oxides on the surface (spectra not shown). Sputtering with 500 eV argon ions reduces the oxide layer of ~30 ÷ 60 Å thick, thereby inducing a Sn^4+^ to Sn^2+^ transformation and the appearance of metallic tin (Sn^0^). In order to elucidate the oxidation state of Sn by laser irradiation, we recorded the XPS valence band spectra (VB) shown in Figure 3b. The most notable difference in the number of the structures between SnO and SnO_2_ oxides (three for SnO_2_ and four for SnO) [13] is in the presence of a prominent peak characteristic of SnO, i.e. the gradual appearance of the Sn 5s – derived structure at low E (2.6 eV) side of the VB after 1800 s and 5700 s ion sputtering. This confirms, the structure of our samples having SnO_2_-SnO-Sn, which is a p-n diode [14].

**Figure 4.**
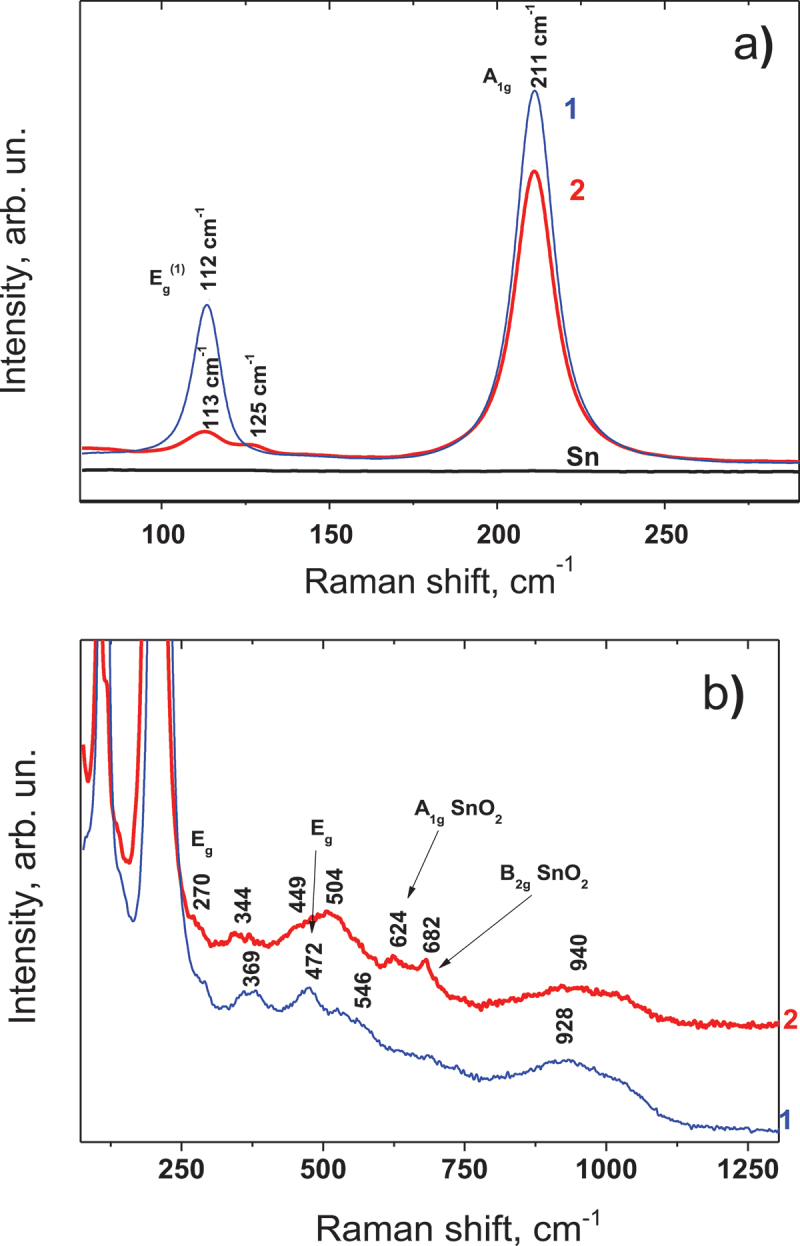
Raman spectra of the SnO layer, formed by pulsed laser processing from different fluence of F = 1.27 J/cm^2^ of the Sn surface in atmospheric conditions in different spectral ranges: (a) 75–290 cm^−1^: (b) 85–1200 cm^−1^.

Unlike the SnO_2_ crystal structure, which belongs to rutile, in SnO the structure is layered and belongs to the P4/nmm space group (D_4 h_^7^ point group) [15]. According to the selection rules for crystalline tetragonal bulk SnO, four vibrational modes may appear in the Raman spectrum [16]. Two of them (A_1 g_, B_1 g_) are polarized along the c-axis, and the other two vibrational modes E_g_ are polarized inside the layers, which are perpendicular to the c-axis. The two most intense bands in our experimental spectrum, with frequencies of 113 and 211 cm^−1^ (Figure 4a) belong to the E_g_^(1)^ and A_1 g_ vibrational modes [2]. Two other, experimentally registered bands with frequencies of 369 and 474 cm^−1^, belong to vibrational modes B_1 g_ and E_g_^(2)^ [2], respectively, and have an intensity almost 2 orders of magnitude lower (Figure 4b). It should be noted that in the Raman spectrum of point 1, the bands from the SnO_x_ and SnO_2_ phases are almost invisible (only low-intensity broad bands in the spectral regions of 520 and 690 cm^−1^, Figure 4b). At the same time, the Raman spectrum of point 2 shows bands from the SnO_x_ intermediate phase (bands with frequencies 125 and 175 cm^−1^, Figure 4a) and bands from SnO_2_ (bands with frequencies 475 (E_g_), 632 (A_1 g_), and 690 cm^−1^, Figure 4b). It is not easy to estimate the average size of the formed SnO nanocrystals from the Raman spectra, since asymmetry is practically not observed for the band with a frequency of 211 cm^−1^, and the latter indicates that the size of the crystals can reach several tens of nanometers. The frequency position of the 632 cm^−1^ (A_1 g_) band, which is somewhat smaller than that of a bulk SnO_2_ crystal, indirectly indicates the size of the SnO_2_ nanocrystals. According to the paper [17], in which the phonon spatial correlation model [18] was used, this frequency position corresponds to the average size of ~45 nm, similar to Raman scattering data. Thus, we create a polycrystalline smooth SnO layer consisting of well-fused nanocrystals.

**Figure 5.**
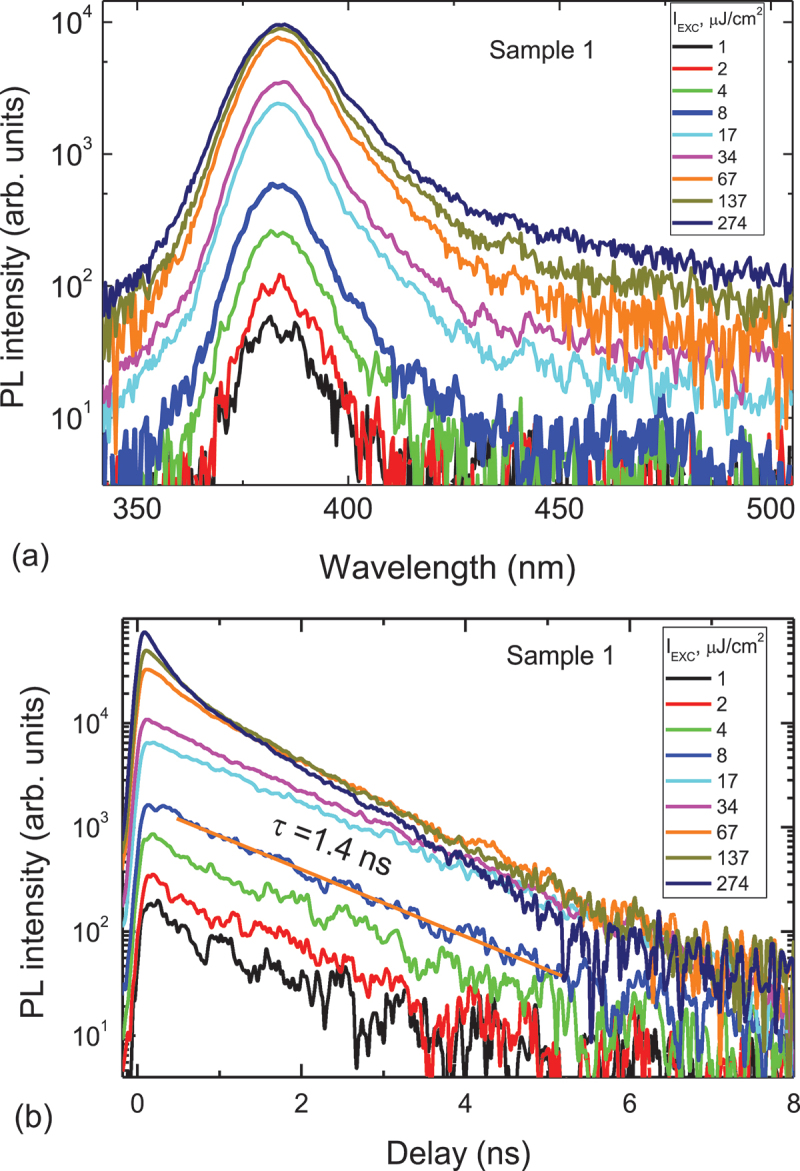
A) PL spectra evolution of the SnO surface depending on the laser excitation fluence. b) Exciton PL decay curves of the SnO layer versus laser excitation energy density.

The photoluminescence spectra are shown in Figure 5a). A wide non-symmetrical emission band with a half width of ~18 nm and maximum at λ ~ 380 nm is observed. The growth of the band long-wave part with an increase of the pump fluence may be connected with the down-conversion of the emitted photons due to re-radiating by the lower energy defect centers (photon transfer) which is planned to be considered in the next study.

**Figure 6.**
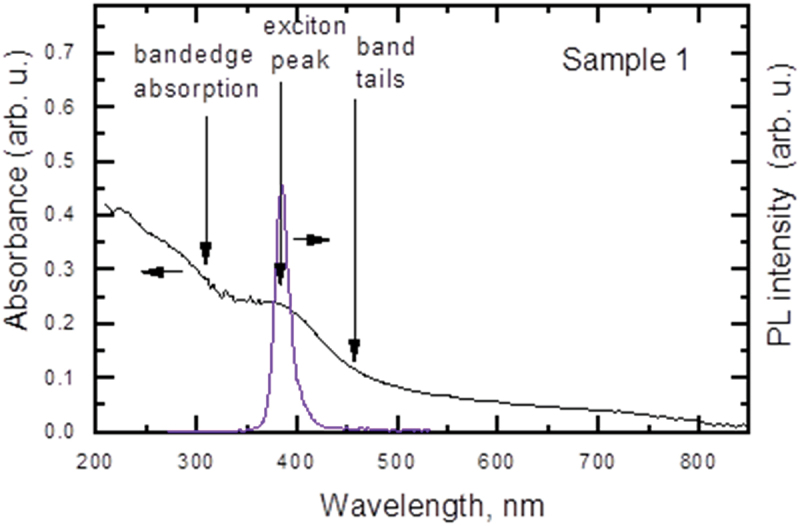
Absorbance and PL spectra of the SnO film.

Samples with the highest XRD SnO peaks show the best exciton emission at 380 nm (3.25 eV) with the longest decay time (1.4 ns, Figure 5b) and the weakest defect emission at 450 nm. The exciton peak position well coincides with the observed exciton absorption peak (Figure 6) which is absent in amorphous phases at non- optimal irradiation conditions. On the other hand, in the defected samples band-tails lead to the defect emission of carriers at lower energies. Higher SnO content, determined by XRD, correlates with the exciton emission enhancement and their lifetime increase due to reduced defect density (Table 1).

**Figure 7.**
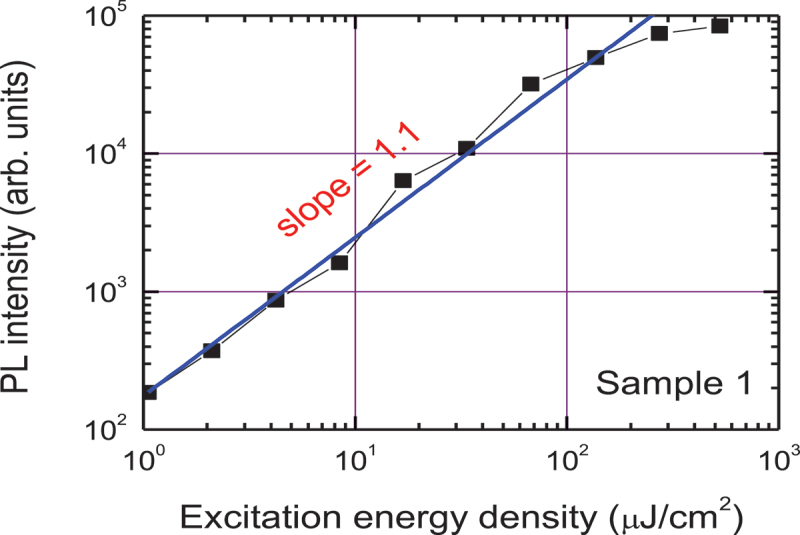
PL exciton band intensity at 380 nm wavelength versus laser excitation energy density.

The optimal laser irradiation treatment shows the best exciton emission at 380 nm and no defect emission in the full excitation range (Figure 5). The exciton peak wavelength is found to be excitation independent. Exponential decay curves at low excitation indicate high homogeneity of the layer and low density of defects; the largest exciton lifetime of 1.4 ns is observed in the sample 1 (Figure 5b). In comparison, the band-edge emission lifetime in SnO_2_ is 1.31 ns [19]. The PL slope is close to unity at low excitation, which can explain the exciton emission (Figure 7).

Using the SnO static dielectric constant ɛ_s_ = 6.2 [20] and the reduced electron–hole effective mass µ = 0.27 m_0_ [21], we calculate the exciton-binding energy E_x_ = 96 meV by using the relation E_x_ = Rµ/ɛ_s_^2^, where *R* = 13.6 eV (SnO_2_ shows similarly large 130 meV exciton binding energy [22]). Such a high binding energy of excitons in SnO indicates that electrons and holes are instantly bound to excitons after excitation similarly as in diamond with 80 meV binding energy [23]. For such a high exciton binding energy, the exciton Bohr radius is a_B_ = a_H_×ɛ_s_×m_0_/µ = 1.2 nm (here a_H_ = 53 pm is the hydrogen atom Bohr radius), which is much smaller than the layer thickness, indicating that the exciton confinement has no considerable impact on its energy. At the highest excitation (>100 µJ/cm^2^), the decays become faster and their amplitude saturates due to the exciton ionization above the Mott transition. At 100 µJ/cm^2^ excitation, we estimate the excited carrier density *N* = 1.1 × 10^19^ cm^−3^ (*N* = αF/(2 hν) [23]. F is the excitation fluence, hν is the quantum energy at 266 nm, and α = 1.6 × 10^5^ cm^−1^ is the absorption coefficient at 266 nm, calculated using the absorbance (provided in the optical density units) in Figure 6 and the layer thickness (d = 47 nm). This critical density agrees well with the Mott density N_Mott_ = 9 × 10^18^ cm^−3^ (calculated according relation 0.25 = a_B_N_Mott_^1/3^).

At the highest excitation, the decays become faster due to the exciton-exciton annihilation and their amplitude saturates due to the exciton ionization above the Mott transition. SnO is typically a p-type semiconductor with p_0_ = 10^18^-10^19^ cm^−3^ hole density, thus a PL superlinear increase of the exciton PL band (380 nm) intensity versus laser pump fluence with a slope of 1.1 indicates light enhancement in the fluence region from ~ 1 to 100 µJ/cm^−2^.

## 4. Conclusions

We produce high-quality polycrystalline SnO nanolayers on Sn by laser irradiation. Irradiation in air was found to be more favorable for SnO formation, as compared to de-ionised water as confirmed by Raman, XRD, XPS, SEM, and PL measurements. This can be explained by the effect of a more non-equilibrium process, which is caused by additional dynamic pressure due to the boiling of water on the tin surface heated by the laser pulse. This can lead to a decrease in the phase explosion threshold and surface destruction. Large exciton lifetime (1.4 ns), determined after femtosecond laser excitation, and no defect emission in sub-micrometer crystallites indicate the applicability of the laser method for the production of topologically defined SnO layers, which can be applied to UV light emitting devices, p-n diodes, and photocatalysts.

## Data Availability

The data that support the findings of this study are available upon reasonable request from the authors.
